# Stress cardiomyopathy after live donor liver transplantation: incidence, risk factors and mortality

**DOI:** 10.1186/cc10794

**Published:** 2012-03-20

**Authors:** S Gupta, D Govil, S Bhatnagar, S Patel, S Srinivasan, P Pandey, M Sodhi, J KN, P Singh, S Saigal, A Soin, V Vohra, Y Mehta

**Affiliations:** 1 Medanta - The Medicity, Gurgaon, India

## Introduction

The incidence of cardiac complications in the post live donor liver transplantation (LDLT) period has been reported to be nearly 70% [1]. Stress cardiomyopathy (SC) is a severe complication which has varied presentation and has grave prognosis if not diagnosed and managed aggressively.

## Methods

Data for 250 LDLTs (June 2010 to July 2011) were collected to assess incidence, risk factors and mortality due to SC. Diagnostic criteria [2] for SC were taken as: global hypokinesia or new ST segment elevation or T-wave inversion in absence of coronary artery disease (CAD) or pheochromocytoma. Etiologies of chronic liver disease and preoperative cardiac status along with intraoperative vasopressor use and dosages were noted.

## Results

**Figure 1:**
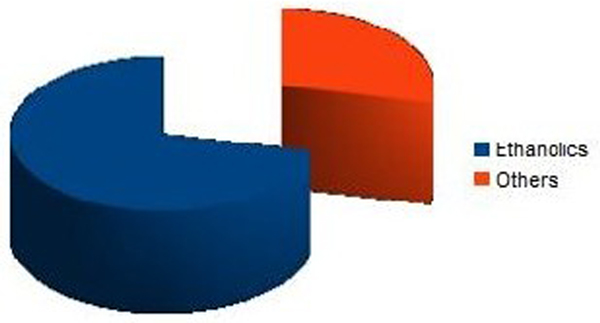


Out of 250 patients five patients had preoperative CAD and were excluded. Seven patients (incidence 2.8%) were diagnosed to have SC. Five out of seven (71.4%) patients were ethanolic and vasopressor requirement was high in all these patients (Figure 1). Echocardiography revealed global hypokinesia with left ventricular ejection fraction between 10 and 25%. They were managed with inotropic support and four patients required an intraaortic balloon pump (IABP). Two patients succumbed to cardiogenic shock on the second day (mortality 28.5%). IABP was weaned between 7 and 9 days. Patients had normal cardiac status at the time of discharge around the fourth week post liver transplant.

## Conclusion

Our incidence was 3%. SC generally presents on the second to third postoperative day and usually recovers by the second week. Ethanolics and patients who require high vasopressor support intraoperatively are more prone to develop SC.
